# A Prader–Willi locus lncRNA cloud modulates diurnal genes and energy expenditure

**DOI:** 10.1093/hmg/ddt281

**Published:** 2013-06-13

**Authors:** Weston T. Powell, Rochelle L. Coulson, Florence K. Crary, Spencer S. Wong, Robert A. Ach, Peter Tsang, N. Alice Yamada, Dag H. Yasui, Janine M. LaSalle

**Affiliations:** 1 Medical Microbiology and Immunology, Genome Center, MIND Institute, University of California, Davis, CA 95616, USA; 2 Agilent Laboratories, Agilent Technologies, Santa Clara, CA 95051, USA

## Abstract

Prader–Willi syndrome (PWS), a genetic disorder of obesity, intellectual disability and sleep abnormalities, is caused by loss of non-coding RNAs on paternal chromosome 15q11-q13. The imprinted minimal PWS locus encompasses a long non-coding RNA (lncRNA) transcript processed into multiple *SNORD116* small nucleolar RNAs and the spliced exons of the host gene, *116HG*. However, both the molecular function and the disease relevance of the spliced lncRNA *116HG* are unknown. Here, we show that *116HG* forms a subnuclear RNA cloud that co-purifies with the transcriptional activator RBBP5 and active metabolic genes, remains tethered to the site of its transcription and increases in size in post-natal neurons and during sleep. *Snord116del* mice lacking *116HG* exhibited increased energy expenditure corresponding to the dysregulation of diurnally expressed *Mtor* and circadian genes *Clock*, *Cry1* and *Per2*. These combined genomic and metabolic analyses demonstrate that *116HG* regulates the diurnal energy expenditure of the brain. These novel molecular insights into the energy imbalance in PWS should lead to improved therapies and understanding of lncRNA roles in complex neurodevelopmental and metabolic disorders.

## INTRODUCTION

Prader–Willi syndrome (PWS), one of the leading genetic causes of obesity in children (1), is characterized by intellectual disabilities, hyperphagia, sleep disorders and increased risk for psychoses and autism (2). PWS is an imprinted disorder caused by paternal deletions of human chromosome 15q11-q13, whereas reciprocal maternal deletions cause the distinct neurodevelopmental disorder Angelman syndrome (3). Parental imprinting is a non-Mendelian inheritance pattern influenced by the sex of the parent because of gametic differences in epigenetic marks such as DNA methylation (4). Human chromosome 15q11-q13 contains multiple maternally silent, paternally expressed protein-coding genes and non-coding RNAs regulated by an imprinting control region (ICR) (5), which when deleted is sufficient to cause PWS (6).

**Figure 1.**
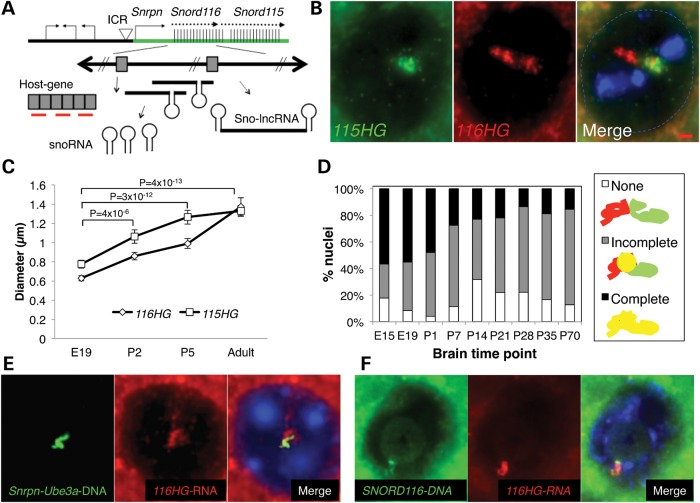
*116HG* and *115HG* are developmentally regulated and form overlapping but distinct nuclear RNA clouds. (**A**) Schematic representation of the murine PWS locus. RNA FISH probes (red) and the DNA FISH probe (green) are shown. (**B**) RNA FISH for *115HG* (green) and *116HG* (red) on adult WT mouse cortex. 4′,6-diamidino-2-phenylindole (blue), scale bar is 1 µm. (**C**) Diameters of RNA cloud signals on individual cortical sections from E19, P2, P5 and adult mice. (**D**) Co-localization of *116HG* and *115HG* clouds. (**E**) Combined DNA and RNA FISH to adult mouse cortical tissue revealed partial co-localization of the *116HG* lncRNA (red) with the paternal decondensed allele of *Snrpn* through *Ube3a* (green). (**F**) Combined DNA and RNA FISH on human cerebellum from a neurotypical female using human-specific RNA and DNA probes (see also Supplementary Material, Fig. S1).

Human genetic studies have defined the minimal functional PWS gene locus to a cluster of non-coding RNAs within the *HBII-85/SNORD116*, making PWS the first human disease found to be caused by loss of non-coding RNA (7–9). The *SNORD116* region encodes repeated C/D box small nucleolar RNAs (snoRNAs) (*SNORD116*) and a spliced long non-coding RNA (lncRNA) host gene (*116HG*) that is stably retained in the nucleus (10) (Fig. 1A). In human cells, an alternative RNA species (*sno-lncRNA*) was recently described that binds the splicing factor Fox2 (11), although the presence in mice of the *sno-lncRNA* has not been confirmed and the relevance to the phenotype of PWS is currently unknown. Mice with an engineered deletion of the *Snord116* repeat cluster (*Snord116del*) recapitulate the PWS phenotype of altered metabolism and growth deficiency, although not obesity (12,13). In mice, the *Snord116/116HG* transcript is expressed only in neurons, suggesting that the metabolic phenotype is due to the central dysregulation of energy use in the central nervous system.

While human and mouse genetics demonstrate the critical nature of the *SNORD116* locus in PWS, the presence of multiple processed RNA products complicates a mechanistic understanding. *SNORD116/Snord116* repeats are adjacent to a set of repeats called *SNORD115/Snord115*, encoding both *115HG* and *SNORD115* RNA products. Both loci are transcribed as part of the same primary transcript originating from the ICR, but only loss of the *SNORD116* region encoding the *116HG* lncRNA is sufficient to cause PWS (7–9). While the *SNORD115* snoRNA functions to regulate alternate splicing of a serotonin receptor (14), the role of the spliced lncRNA *116HG* has not been investigated.

LncRNAs exert essential functions via diverse mechanisms, including scaffolds for chromatin remodeling complexes or decoys for nucleotide binding (15,16). Although many lncRNAs such as *116HG* are expressed in the brain, their role in human disease, including PWS, is uncharacterized (17). Interestingly, over 50 C/D box snoRNA host genes were unexpectedly identified in a recent genomic screen for diurnally regulated transcripts in *Drosophila* (18), but the functional relevance of snoRNA host genes in diurnal transcription is completely unknown.

In this study, we use a combination of *in situ* molecular genetics, genomic and whole-body metabolism approaches to functionally characterize the lncRNA *116HG* that emerges in the brain in the first week of life as an RNA cloud in neuronal nuclei. The transcriptional activator RBBP5 and 2403 active metabolic genes were identified as significantly associated with *116HG* during the light phase, when *Snord116del* mice exhibited dysregulation of diurnally expressed *Mtor* and circadian genes *Clock*, *Cry1* and *Per2.* Genome wide, over twice as many genes in the light compared with the dark phase, showed altered expression in *Snord116del* versus wild-type (WT) mice, corresponding to the metabolic phenotype of the reduced respiratory exchange ratio (RER) and the increased energy expenditure in this PWS mouse model.

## RESULTS

### *116HG* forms a developmentally regulated lncRNA cloud

To understand the expression and localization of the *116HG* lncRNA in mammalian brain development, RNA fluorescence *in situ* hybridization (FISH) using probes targeting the splice junction of *116HG* or *115HG* (Fig. 1A) was performed on mouse brain tissue, revealing two distinct cloud-like nuclear domains in adult neuronal nuclei (Fig. 1B). In contrast, *Snord116del* mouse brain showed the *115HG*, but not the *116HG*, lncRNA cloud (Supplementary Material, Fig. S1). The *116HG* and *115HG* nuclear clouds observed were similar to small ‘grape’ structures seen previously in primary neuronal cultures (10), but covered a much larger area of the nucleus in adult brain neurons. *116HG* and *115HG* nuclear clouds were observed in neurons but not glia throughout brain regions of hypothalamus, hindbrain, forebrain, cortex, hippocampus and cerebellum, but not in cells of the liver or spleen (data not shown). Both *116HG* and *115HG* lncRNA clouds significantly increased in diameter during the first week of post-natal life (Fig. 1C), and co-localization analysis revealed that *116HG* and *115HG* separated into overlapping but distinct lncRNA clouds in the post-natal cortex (Fig. 1D). The increased size and separation of *116HG* and *115HG* lncRNA clouds developmentally coincided with the previously described nucleolar accumulation of *Snord116* snoRNAs and the large-scale chromatin decondensation of the paternal *Snrpn-Ube3a* locus containing the *Snord116* repeat cluster (19).

Combined RNA and DNA FISH on the adult cortex revealed that *116HG* and *115HG* localized to the paternal decondensed allele of *Snrpn-Ube3a* (Fig. 1E and Supplementary Material, Fig. S1). The *Snord116* DNA overlapped with *116HG*, but not *115HG*, and the two DNA loci (*Snord116* and *Snord115*) co-localized in adult kidney, but not brain, nuclei (Supplementary Material, Fig. S1) consistent with the neuronal tissue-specific chromatin decondensation of this locus (19). Human-specific RNA/DNA FISH probes confirmed that *116HG* forms an RNA cloud localized to the decondensed *SNORD116* paternal allele in postmortem human brain (Fig. 1F), showing the relevance of the *116HG* lncRNA cloud for understanding human PWS.

### *116HG* binds to RBBP5 and target loci genome wide

**Figure 2.**
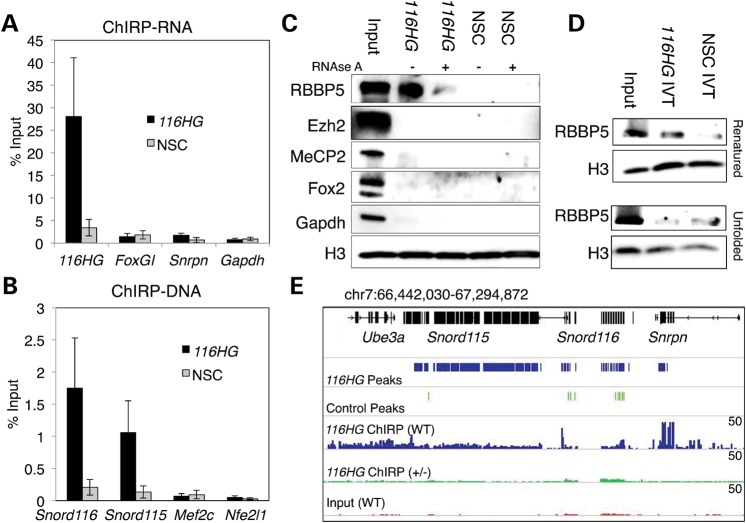
*116HG* RNA interacts with RBBP5 and its endogenous locus. (**A**) qRT-PCR analysis of ChIRP RNA shows retrieval of *116HG* lncRNA with a *116HG*-specific probe, but not the NSC probe. (**B**) qPCR analysis of ChIRP DNA shows retrieval of *Snord116* DNA with a *116HG*-specific probe, but not the NSC probe. (**C**) Western blotting of ChIRP proteins shows that *116HG* retrieved RBBP5 in an RNA-dependent manner, but not MeCP2, Fox2 or Gapdh. (**D**) Pull-down of IVT biotinylated *116HG* retrieved RBBP5 when allowed to renature under slow-cooling conditions but less efficiently in the unfolded state. (**E**) *116HG* ChIRP-seq reveals peaks in the *Snord116* and *Snord115* regions, which are absent in NSC ChIRP-seq samples. Top black track represents annotated genes. Blue represents *116HG* ChIRP peaks and read coverage of *116HG* ChIRP from WT brain (scale 0–50 reads). Green represents called peaks for merge of three control ChIRP experiments (*116HG* ChIRP on *Snord116del*^+/−^ brain and NSC ChIRP on WT and *Snord116del*^+/−^ brain) and read coverage from *116HG* ChIRP on *Snord116del*^+/−^ brain. Red represents WT input. qPCR results graphed as the mean ± SEM (see also Supplementary Material, Fig. S2).

Other lncRNAs that form subnuclear RNA clouds, such as *Xist* and *Kcnq1ot1*, bind proteins that regulate transcription of target regions (15,16). In order to isolate *116HG*-associated proteins and interacting DNA from mouse brain, we adapted a method called chromatin isolation by RNA purification (ChIRP) (20–22) using biotinylated antisense oligos complementary to *116HG*. Reverse transcriptase–quantitative polymerase chain reaction (RT–qPCR) on RNA isolated by ChIRP showed 25–30% of input *116HG* RNA was retrieved by *116HG*-specific oligos but not by non-specific control (NSC) oligos (Fig. 2A). Analysis of DNA isolated by ChIRP revealed that *Snord116* DNA was retrieved by *116HG*-specific oligos (Fig. 2B), confirming DNA FISH results (Fig. 1E). Immunoblotting for candidate proteins interacting with *116HG* revealed that RBBP5 (but not MeCP2, Fox2 or Gapdh) was specifically retrieved by *116HG* ChIRP in an RNA-dependent manner (Fig. 2C). RBBP5 is a subunit of the MLL1 complex that activates transcription through methylation of histone H3K4 and interacts with the lncRNA *Mistral* to activate *Hoxa6* and *Hoxa7* during stem cell differentiation (23). An *in vitro* transcribed (IVT), biotinylated *116HG* also retrieved RBBP5 in a conformation-dependent manner, requiring slow-cooling following denaturation (Fig. 2D and Supplementary Material, Fig. S2). An unbiased proteomic screen of *116HG* ChIRP identified the *Xist* associating protein SAF-A(24), although SAF-A association with *116HG* was non-specific and not RNAse sensitive (Supplementary Material, Fig. S2), unlike RBBP5.

Since the large size (>1.2 μm) of the *116HG* lncRNA nuclear subdomain suggested that *116HG* may interact in *trans* with other gene loci, DNA isolated by ChIRP was analyzed by next-generation sequencing (ChIRP-seq). Sequencing libraries were made from ChIRP DNA samples from adult WT or *Snord116del*^+/−^ mouse brain using *116HG*-specific or NSC oligos. As expected based on qPCR analysis (Fig. 2D) and FISH (Fig. 1E and Supplementary Material, Fig. S1), *116HG* ChIRP-seq peaks from the WT brain were enriched at the *Snord116* and the *Snord115* region (Fig. 2E) at levels comparable with published ChIRP methods (20).

**Figure 3.**
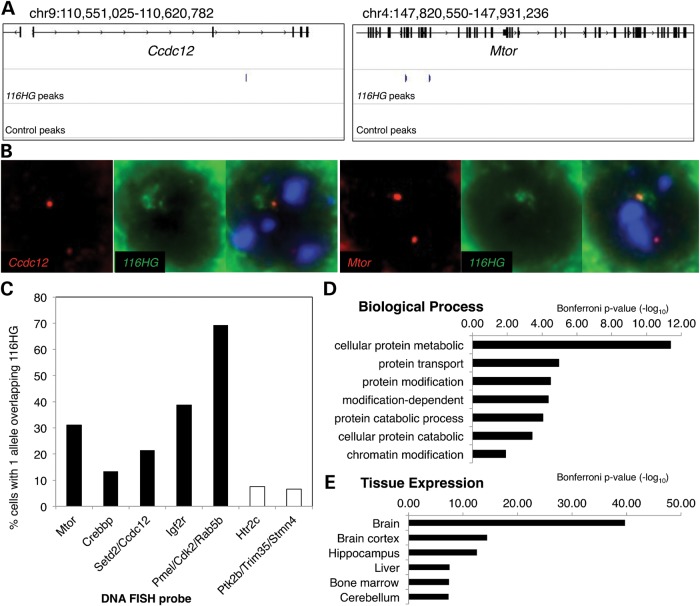
*116HG* RNA binds to loci genome wide. (**A**) *116HG* associates with distant loci. Blue track shows *116HG* ChIRP peaks from the WT brain, while lower track shows the absence of control ChIRP peaks at these gene loci. (**B**) Combined RNA and DNA FISH to adult mouse cortical tissue confirmed co-localization of the *116HG* lncRNA (green) with *Ccdc12* and *Mtor* loci (red). (**C**) Gene loci identified by *116HG* ChIRP (black) showed greater overlap with *116HG* than negative control regions (white). *n* = 695 nuclei. (**D** and **E**) GO analysis of *116HG* ChIRP identified genes (see also Supplementary Material, Fig. S3).

In addition, analysis revealed *116HG*-specific ChIRP peaks associated with 2403 genes (Fig. 3A and Supplementary Material, Fig. S2 and Supplementary Material, Table S1). Combined RNA/DNA FISH experiments confirmed the enriched co-localization of these ChIRP target genes with the *116HG* lncRNA cloud, compared with two negative control regions (Fig. 3B and C). Gene ontology (GO) analysis revealed that the *116HG*-specific ChIRP genes were enriched for brain expression, protein transport, protein and chromatin modifications and protein metabolic processes (Fig. 3D and E and Supplementary Material, Table S2). *116HG*-specific ChIRP genes included the mammalian target of rapamycin (*Mtor*), transcriptional regulator Creb-binding protein (*Crebbp*) and imprinted insulin growth factor receptor (*Igf2r*).

### Loss of *116HG* leads to up-regulation of genes

**Figure 4.**
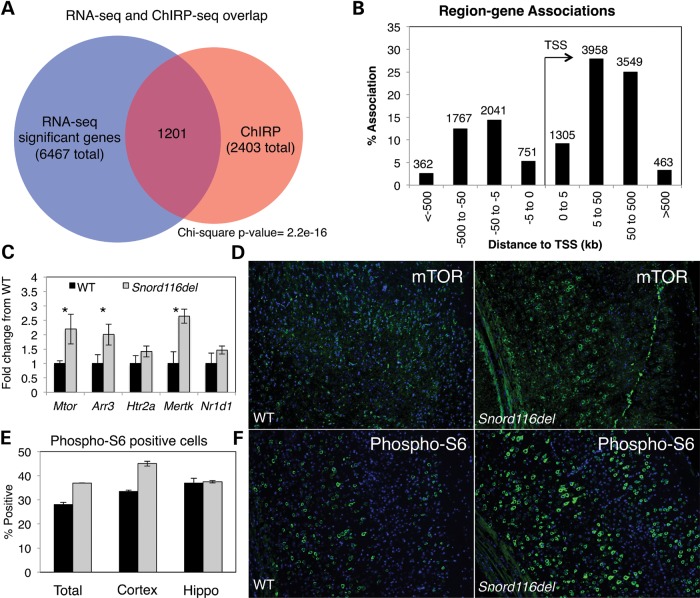
Combined analysis of *116HG* ChIRP-seq with RNA-seq of WT versus *Snord116del* cortex reveals up-regulation of *116HG* bound genes including *Mtor*. (**A**) Genes associated with *116HG*-specific ChIRP peaks overlapped significantly with genes showing transcript level changes by RNA-seq. (**B**) *116HG* binding sites show clustering 5–500 kb up or downstream from transcription start sites. (**C**) qRT-PCR confirms significant up-regulation of 3/5 genes identified by RNA-seq: *Mtor* (*P* = 0.042), *Arr3* (*P* = 0.049), *Mertk* (*P* = 0.007) in a larger set of WT (*n* = 6) and *Snord116del* (*n* = 12) mouse cortex. (**D**) Immunofluorescence staining of WT and *Snord116del* prefrontal cortex for mTOR showing increased expression in *Snord116del*. (**E**) Laser scanning cytometry quantitation of phospho-S6 staining of WT (black, *n* = 3) compared with *Snord116del* (grey, *n* = 3) for total brain slice, cortex or hippocampus. (**F**) Immunofluorescence staining of WT and *Snord116del* cortex for phospho-S6.

We investigated the effect of loss of *116HG* on transcript levels genome wide by comparing WT versus *Snord116del*^+/−^ mice by RNA-seq analysis of the adult cortex. 6467 genes were significantly altered (*q*-value of <0.05) in the *Snord116del* compared with the WT cortex, with 94.2% up-regulated in the *Snord116del* cortex (Supplementary Material, Table S3). Comparison of *Snord116del* dysregulated genes to ChIRP-seq peaks revealed that 50% (1201) of genes identified by ChIRP had altered expression levels in the *Snord116del* cortex (Fig. 4A and Supplementary Material, Table S4). GO analysis of genes identified by ChIRP and RNA-seq analysis was enriched for protein and cellular metabolic processes, chromatin modification, cellular biosynthetic processes, neurogenesis and central nervous system development (Supplementary Material, Table S5). Most ChIRP peaks were located within genes or distal to transcription start sites (TSSs) (Fig. 4B), suggesting that *116HG* association with target genes may be sequestering RBBP5 away from promoter regions. qRT-PCR analysis confirmed the up-regulation of genes identified by RNA-seq analysis in an independent set of mice (Fig. 4C). Combined, these results suggest a decoy model in which *116HG* prevents RBBP5 binding to promoters of *116HG*-associated genes, similar to that described for lncRNA *Gas5* and the glucocorticoid receptor (25).

The overlap of ChIRP-seq and RNA-seq results included genes important for signal transduction and protein catabolism, including the metabolic regulator *Mtor*. To further investigate changes in mTOR signaling, WT and *Snord116del^+/−^* brain slices were immunostained for mTOR protein and for phospho-S6, a downstream target of mTOR (26) (Fig. 4E and F). The mTOR signal was increased and significantly more cells were positive for phospho-S6 in the *Snord116del* cortex compared with WT littermates (Fig. 4E), confirming increased mTOR activity. mTOR up-regulation appeared to be cortex-specific, as phospho-S6 staining was unchanged in *Snord116del* hippocampus (Fig. 4E).

### *Snord116del* mice show alteration of diurnally regulated genes

**Figure 5.**
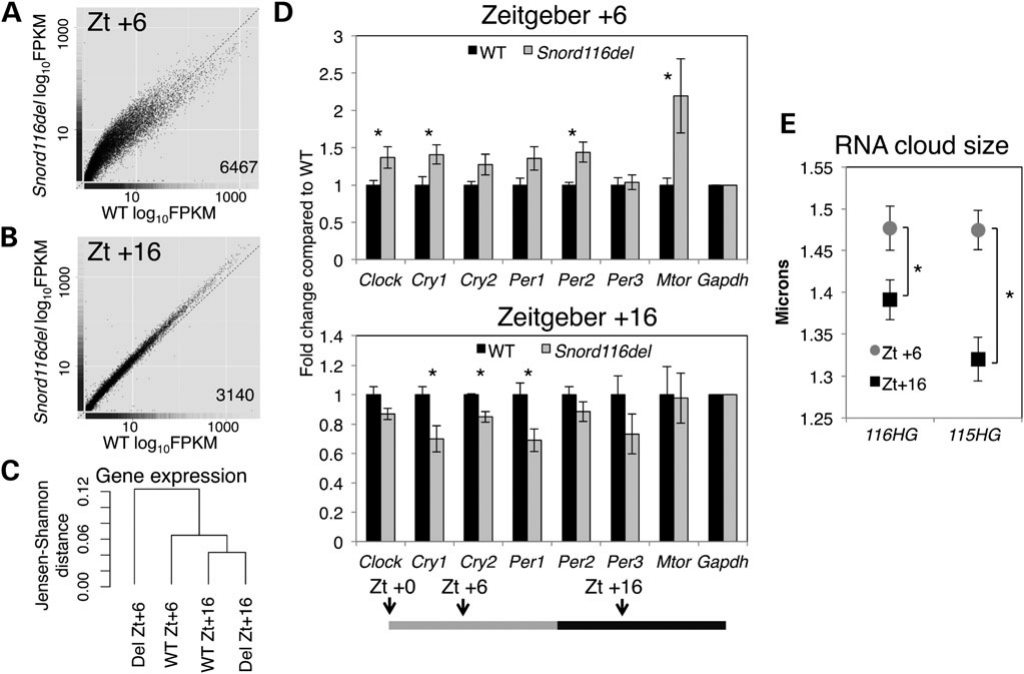
*Snord116del* mice show altered transcript levels of diurnally regulated genes and RER during light hours. (**A**) RNA-seq shows up-regulation of many genes at Zt +6 in *Snord116del* versus WT cortex, and (**B**) fewer genes changed at Zt +16 in *Snord116del* versus WT cortex. The number of significant genes labeled in each graph. (**C**) Dendrogram-based analysis of gene expression from samples in RNA-seq analysis. (**D**) Diurnally regulated genes were up-regulated at Zt +6 and down-regulated at Zt +16 in *Snord116del* versus WT cortex. Transcript levels were normalized to *Gapdh*, and fold change from WT graphed as the mean ± SEM. For Zt +6 timepoint, *n* = 6 WT, 12 *Snord116del*^+/−^. For Zt +16, *n* = 5 WT, 6 *Snord116del*^+/−^. Zt +6 *Clock* (*P* = 0.037), *Cry1* (*P* = 0.035), *Per2* (*P* = 0.010), *Mtor* (*P* = 0.042). Zt +16 *Cry1* (*P* = 0.019), *Cry2* (*P* = 0.007), *Per1* (*P* = 0.022). (**E**) *116HG* (*P* = 0.016) and *115HG* (*P* = 1.3 × 10^−05^) lncRNA clouds are significantly smaller at Zt +16 compared with Zt. +6. *n* = 720 nuclei and 3 biological replicates per sample (see also Supplementary Material, Fig. S3).

mTOR activity is controlled by a circadian clock in the suprachiasmatic nucleus (26), and a prior study in WT C57/BL6 mice revealed many cortical genes to be diurnally regulated (27). Therefore, we investigated whether *Snord116del* loss had an effect on transcription associated with diurnal time. WT and *Snord116del* littermates were sacrificed at Zeitgeber (Zt) +6 h and Zt +16 for RNA-seq analysis of cortical transcripts. At Zt +6, 6467 genes were altered in the *Snord116del* cortex (Fig. 5A), but at Zt +16 only 3140 transcripts changed (Fig. 5B and Supplementary Material, Table S6). Genes altered in the *Snord116del* cortex at Zt +6 significantly overlapped with genes previously described to be diurnally regulated (27) as well as genes identified in our analysis as significantly changed in WT mice at Zt +6 compared with Zt +16 (Supplementary Material, Fig. S3). Dendrogram analysis of gene expression revealed that WT and *Snord116del* from Zt +16 were more closely related than WT and *Snord116del* from Zt +6 (Fig. 5C). qRT-PCR confirmed the significant increase in diurnally regulated *Cry1*, *Clock*, *Per2* and *Mtor* transcripts specifically at Zt +6 (Fig. 5D). Furthermore, *116HG* and *115HG* lncRNA clouds were significantly smaller in the cortex of WT mice at Zt +16 when compared with Zt +6, revealing that these nuclear lncRNA subdomains dynamically change with diurnal time (Fig. 5E).

### *Snord116del* mice are lean and have increased lipid oxidation during light hours

**Figure 6.**
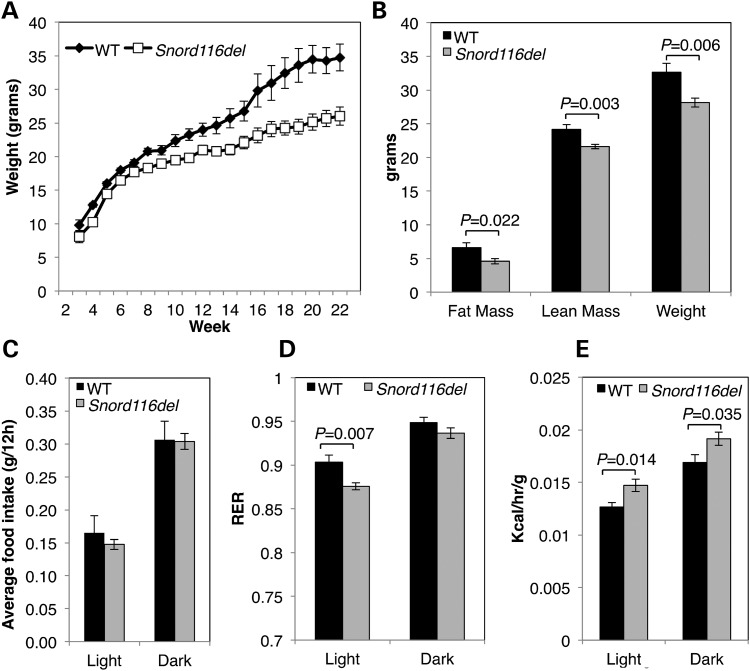
*Snord116del* mice are leaner than WT littermates and have increased lipid oxidation during light hours. (**A**) *Snord116del*^+/−^ gain weight at a slower rate than WT littermates and (**B**) are smaller as adults corresponding to decreased fat mass, lean mass and total body weight. (**C**) *Snord116del*^+/−^ do not have altered food intake compared with WT littermates during light or dark hours. (**D**) *Snord116del*^+/−^ mice exhibit lower RER during light hours when compared with WT mice. (**E**) *Snord116del*^+/−^ mice have increased energy expenditure during light and dark hours (see also Supplementary Material, Fig. S4). Results are shown for male mice (*n* = 14 per group in A; WT, *n* = 12; *Snord116del*, *n* = 14 for B–E).

We sought to relate altered diurnal transcript levels to the metabolic phenotype of adult *Snord116del* mice (12), which were previously shown to have lower body weight, fat mass and lean mass compared with WT littermates (Fig. 6A and B). Mice were continuously monitored in Comprehensive Lab Animal Monitoring System (CLAMS) metabolic chambers for a period of 6 days in order to measure their metabolism, food intake and activity. *Snord116del* mice did not differ from WT littermates in their food intake or activity (Fig. 6C and Supplementary Material, Fig. S4). However, *Snord116del* mice exhibited a significant decrease in the RER compared with WT littermates during light hours (Fig. 6D and Supplementary Material, Fig. S4). The RER reflects the relative source of energy for the animal with values closer to 1 representing increased carbohydrate oxidation and values closer to 0.7 representing increased fat oxidation. An increase in the RER occurs during dark compared to light hours in both WT and *Snord116del* mice (Fig. 6D), consistent with increased food intake and activity during dark hours (Supplementary Material, Fig. S4). In contrast to WT mice, *Snord116del* mice have decreased RER during light hours, which explain the decreased fat mass and body weight in *Snord116del* mice (Supplementary Material, Fig. S4). *Snord116del* mice also showed increased energy expenditure compared with WT mice during both light and dark hours (Fig. 6E). However, energy expenditure is dependent on body weight, and analysis of covariance revealed that the increased energy expenditure was not independent of body weight. During light hours, WT mice reduce their energy expenditure, decrease catabolism and increase anabolism (28). *Snord116del* did show the expected diurnal cycling of their RER, energy expenditure, activity and feeding (Fig. 6C–E and Supplementary Material, Fig. S4), suggesting that their central circadian clock is not disrupted and that the metabolic phenotype is due to the central dysregulation of energy expenditure, not changes in feeding or activity. Our results suggest that a key feature of the *Snord116del* phenotype is altered the regulation of diurnal energy source due to loss of *116HG* modulation of diurnally regulated circadian and metabolic genes during light hours.

## DISCUSSION

While the loss of the *Snord116* locus was first hypothesized 10 years ago to be responsible for the pathogenesis of PWS (29), research has focused on the roles of the orphan snoRNAs, *SNORD116* and *SNORD115*, and editing of the serotonin receptor (*5htrc*) by *SNORD115* (14). However, our results show that the flanking snoRNA host exons form a functional lncRNA structure, *116HG*, which regulates the transcription of genes important for the regulation of diurnal transcription and metabolism. Our work, therefore, provides a novel explanation that loss of a specific lncRNA alters circadian energy homeostasis in PWS. In humans, the *SNORD116* locus gives rise to *sno-lncRNA* in addition to *116HG* (11). Although arising from the same primary transcript, *116HG* and *sno-lncRNA* act via independent mechanisms. In our studies, we used an RNA FISH probe spanning an exon–exon boundary that is not present in *sno-lncRNA* and did not observe an interaction of Fox2 with *116HG* (Fig. 2C). Furthermore, there is no evidence within the mouse genomic sequence for the existence of *sno-lncRNA*, suggesting that the metabolic phenotype seen in the *Snord116del* mice is due to loss of *116HG*. However, future studies will need to identify which of the RNAs generated from the *Snord116* locus are critically lost in PWS, and if loss of multiple RNAs underlies the complex phenotype of PWS.

Sleep problems in PWS patients, including longer latency and shorter duration of night-time sleep and excessive daytime sleepiness, are consistent with a disruption in circadian metabolism being central to the disease phenotype, rather than a result of obesity (30). A mouse model of *Magel2* deficiency, a gene missing in PWS patients with large deletions, also showed deficits in circadian rhythms (31), but the observations reported here link circadian imbalances in the PWS critical locus mouse model directly to loss of a lncRNA. The identification of over 50 C/D box snoRNA host genes of unknown function identified in a *Drosophila* screen for diurnally regulated transcripts (18) suggests that the diurnal regulation of an snoRNA host gene is evolutionarily conserved. Since lncRNAs can act to regulate the transcription of domains of multiple genes (16), *116HG* may coordinate the transcriptional levels of multiple genes that have diurnal variation in expression. In this study, we have identified a group of 1201 genes that are both significantly associated with *116HG* and significantly up-regulated in the absence of *116HG*, suggesting a direct transcriptional regulation by the *116HG* cloud during the light phase.

Disruption of sleep and diurnal rhythms of feeding in mice and humans is associated with metabolic disorders that can lead to either increased weight gain (32) or protection from obesity (33). While hyperphagia in PWS is a key aspect of the phenotype and contributes to a large extent to the obesity, infants with PWS do not have increased caloric intake, but they do have increased weight gain (34). A previous study of the *Snord116del* mice found that they had increased food intake when compared with WT littermates (12); however, in that study, food intake was normalized to body weight and the relative increase was dependent on lower body weights in *Snord116del* mice. In our study, we found no evidence for hyperphagia in *Snord116del* mice relative to body weight or lean mass. Instead, we found that *Snord116del* mice do not differ from WT mice in their food intake and that the lean phenotype that has been reproduced across two mouse models (12,13) is driven by dysregulated energy expenditure and metabolic fuel source. This finding indicates that a key mechanism in the pathogenesis of PWS is loss of energy balance due to loss of the non-coding RNAs from the *Snord116* region.

Furthermore, in human PWS, weight increases before the onset of hyperphagia or increased food intake (34), supporting the hypothesis that a primary factor in the onset of obesity in PWS is loss of energy regulation. In mice, diurnal rhythms become established near post-natal day 10 (35). A previous study of gene expression in *Snord116del* mouse hypothalamus found no changes in gene expression during the first 2 weeks of life (36), which could be due to immature establishment of diurnal rhythms at the timepoints analyzed. In contrast, our study investigated the *Snord116del* cortex in adulthood when diurnal rhythms are established and the greatest differences in metabolism and weight are seen when compared with WT littermates. In humans, diurnal rhythms begin to appear at 1 month after birth and become established over the first year of life (37). The establishment of diurnal rhythms during infancy may relate to the phenotypic switch observed in PWS from one of early failure-to-thrive in infancy to later obesity and altered metabolism (34).

## MATERIALS AND METHODS

### Animal care

B6(Cg)-Snord116^tm1.1Uta^/J (*Snord116del*) mice were obtained from Jackson Labs (Bar Harbor, ME, USA) and housed in a 24-h light/dark cycle (6 a.m.–6 p.m. light, 6 p.m.–6 a.m. dark), temperature controlled room and fed a standard diet of Picolab mouse chow 20 (PMI International, St Louis, MO, USA). Animal genotyping was performed using PCR from tail DNA as described previously (19). Heterozygous deletion male mice were bred with C57BL/6 WT females to generate paternal deletion *Snord116del*^+/−^ and WT littermates.

### Human tissue

Human postmortem cerebellum of brain 1209 was received frozen and was fixed in 10% formalin, embedded and sectioned (5 µm). Tissue was obtained through the NICHD Brain and Tissue Bank for Developmental Disorders located at the University of Maryland School of Medicine.

### Mouse tissue

For RNA, DNA and RNA/DNA FISH analyses, tissue was prepared as described previously (19). For the oligo-based purification of RNA-associated proteins and *in vitro* biotinylated RNA pull-down of proteins, one hemisphere of a WT adult C57BL/6 brain was fixed in 3.7% formaldehyde/1× phosphate buffered saline (PBS) and homogenized. Nuclei were resuspended in RNA immunoprecipitation/formamide buffer [150 mm KCl, 25 mm Tris–HCl, pH 7.5, 5 mm ethylenediaminetetraacetic acid (EDTA), 0.5% NP-40, 10% formamide with Superasin (Ambion) and complete proteinase inhibitors (Roche) added]. For ChIRP, one hemisphere of a WT adult C57BL/6J or *Snord116del* brain was fixed in 3.7% formaldehyde/1× PBS and homogenized. Nuclei were resuspended in ChIRP lysis buffer (50 mm Tris–HCl, pH 7.5, 10 mm EDTA, 1% sodium dodecyl sulfate +Superasin and proteinase inhibitors). For RNA-sequencing analysis, the brains from adult *Snord116del* and WT littermates were collected and cortices were dissected and placed in RNALater (Ambion) before RNA isolation.

### RNA FISH and DNA FISH

FISH was performed as described previously (19). Overlap of RNA FISH clouds was analyzed by first outlining the area of *115HG* and then scoring for complete, partial or no signal from *116HG* within the area defined by *115HG*. DNA FISH measurements were taken using a 100 × oil objective with a 2 × zoom and pixel counts converted to micron distance. All measurements for a given experiment were taken with the same exposure times and microscope settings appropriate for the fluorescence intensity. RNA FISH on human postmortem cerebellum samples was performed using custom directly labeled, single-stranded oligo library synthesis probes (Agilent). Significant changes tested by two-tailed Student's *t*-test.

### RNA/DNA FISH

Slides were prepared as for RNA FISH and hybridized with locked nucleic acid probes (Exiqon) overnight at 46°C and washed 3× in 50% formamide/2× saline-sodium citrate (SSC) and 2× in 2× SSC at 46°C, fixed with 3.7% formaldehyde/1× PBS at RT for 5 min, washed in 1× PBS and dehydrated through a series of ethanol washes (50, 70, 90 and 100%). DNA FISH probes were then applied and slides were denatured for 3 min at 85°C. Washing and detection was performed as for RNA or DNA FISH alone.

### Immunofluorescence and laser scanning cytometry

Immunofluorescence and laser scanning cytometer (LSC) analysis was performed as described previously (19).

### Microscopy

Slides were analyzed on an Axioplan 2 fluorescence microscope (Carl Zeiss, Inc., NY, USA) equipped with a Qimaging Retiga EXi high-speed uncooled digital camera, appropriate fluorescent filter sets and automated xyz stage controls. Images were captured with appropriate filter sets at 0.1 µm sections and multiple Z-slices were combined for final images. For phospho-S6 quantification, slides were scanned on a laser scanning cytometer (CompuCyte) with a 20× objective. Nuclei were contoured using 4′,6-diamidino-2-phenylindole fluorescence, the contours extended by 15 pixels, and cells were gated for fluorescence above background. All slides were scanned on identical voltage, photomultiplier tube and threshold settings. Brain regions were partitioned based on the composite image from all scan fields. Significant changes tested by Student's *t*-test.

### Oligo purification of RNA-bound proteins

200 pmol of biotinylated DNA oligos (Invitrogen) were added to 1 mg of material and incubated at 4°C for 4 h. C-1 streptavidin beads (Invitrogen) were used to retrieve biotinylated DNA oligos with hybridized RNA:protein complexes. Proteins were analyzed by immunoblotting. For RNAse sensitivity, Superasin was omitted and 10 μg of RNAse A (Ambion) was added to sonicated material and incubated at 37°C for 30 min before biotinylated probe was added. For mass-spec identification, proteins were run on an sodium dodecyl sulfate–polyacrylamide gel electrophoresis gel and stained with Imperial protein stain. A 1 cm square piece of gel from 80–140 kDa was excised and submitted to the UC Davis Proteomics core for mass-spec identification.

### *In vitro* biotinylated RNA pull-down of proteins

Template DNA was linearized and 250 ng of DNA was IVT using Biotin RNA labeling Mix (Roche), DNAse I (NEB) treated and nucleotides removed using Micro-Biospin columns (Biorad). IVT RNA was denatured at 65°C for 5 min and then slow-cooled to 4°C. RNA was quantified using a Nanodrop spectrophotometer, 100 pmol RNA was added to 1 mg of lysate and incubated for 2 h at RT. C-1 streptavidin beads were used to retrieve biotinylated RNA and bound proteins were analyzed by immunoblotting.

### Antibodies

Primary antibodies: anti-GAPDH clone 6C5 (Advanced Immunochemical), anti-RBBP5 (Bethyl), anti-MeCP2 (Cell Signaling), anti-FOX2, anti-histone H3-total (Abcam), anti-SAF-A/HNRNP-U (Novus Biologicals) and anti-Ezh2 (Active Motif). Secondary antibodies: GARIG-Alexa-488 (Life Technologies), anti-DIG-Alexa-488, anti-biotin-Rhodamine (Jackson), GARIG-HRP and GAM-HRP (Biorad).

### Chromatin isolation by RNA purification

Brain cell lysate was sonicated on high in a Bioruptor sonicator (Diagenode) at 4°C until solution was no longer turbid and size of fragments was 200–800 bp by gel analysis. Insoluble material was pelleted by centrifugation at 16 000*g* for 5 min at 4°C. 100 μl was saved as input for DNA purification and 100 μl for RNA purification. ChIRP was performed as described previously (20). 10 μl of eluted DNA was used to prepare sequencing libraries using NuGEN Ovation Ultralow Library kit following the manufacturer's instructions for multiplexing with barcodes L2DR-BC1-8.

### RNA-sequencing

RNA was purified from dissected cortices using RNEAsy columns (Qiagen) following the manufacturer's instructions and analyzed on a Bioanalyzer. Samples with an RNA integrity number > 7 were used to prepare libraries using the NuGEN Encore Complete RNA-seq library kit following the manufacturer's instructions for multiplexing, with cDNA fragmentation performed on a Bioruptor for 1 h at 4°C. Three WT males from Zt +6, two WT males from Zt +6, two *Snord116del* males from Zt +6 and two *Snord116del* males from Zt +16 were analyzed.

### Next-generation sequencing

Sequencing libraries for ChIRP-sequencing and RNA-sequencing were analyzed for quality on a Bioanalyzer (Agilent) and were sequenced on a Hi-seq-2000 at the Vincent J. Coates Genomics Sequencing Laboratory at UC Berkeley.

### ChIRP-sequencing data analysis

ChIRP-sequencing reads were aligned using Bowtie2 against the *Mus musculus mm9* genome, and peaks were called using model-based analysis for ChIP-Seq (MACS) (38,39). The numbers of aligned reads out of total reads for each sample were WT-*116HG*-ChIRP-1: 29,814,977/42,110,162; WT-*116HG*-ChIRP-2: 22,522,642/91,706,595; WT-NSC-ChIRP: 29,064,400/41,230,076; *Snord116del*-*116HG*-ChIRP: 38,787,232/40,374,703; *Snord116del-*NSC ChIRP: 48,493,474/50,605,786; Input: 23,704,637/25,610,883. MACS identified peaks from ChIRP were filtered by the false discovery rate (<5%) and fold-enrichment over input (>20). Since no sequence motifs were identified using all the called *116HG* ChIRP peaks from two biological replicates, we used a 10 kb window to identify peaks that appeared in the same genomic region in both WT-*116HG* ChIRP samples. 8105 MACS identified peaks replicated between the two WT-*116HG* ChIRP samples. In contrast, of 8300 MACS-called peaks combined from the three negative control ChIRP samples (WT-NSC, *Snord116del*-*116HG* and *Snord116del*-NSC), there was <0.05% overlap between samples and only 985 peaks were within 10 kb of *116HG*-ChIRP identified peaks. The BED file with corresponding confident peaks was then used to generate an associated gene list by mapping to closest gene within 2 kb of the 8105 replicated peaks. Genes associated with *116HG* peaks were submitted for DAVID GO analysis (40,41). ChIRP peaks were mapped relative to TSS using Genomic Regions Enrichment of Annotations Tool (GREAT) http://bejerano.stanford.edu/ (42).

### RNA-sequencing data analysis

Reads were aligned against the *Mus musculus mm9* genome using Tophat2 with a reference gene transfer format guide. Reads per sample were: WT-Zt6-1 56,801,996, WT-Zt6-2 64,288,201, WT-Zt6-3 63,022,540, *Snord116del*-Zt6-1 53,278,255, *Snord116del*-Zt6-2 40,243,885, WT-Zt16-1 63,985,980, WT-Zt16-2 56,483,862, *Snord116del*-Zt16-1 54,310,542 and *Snord116del*-Zt16-2 61,584,399. Cuffdiff was used to identify significant changes of transcript levels between paternal inherited *Snord116del^+/−^* and WT mice (43) with a *q*-value of <0.05. CummeRbund was used to extract cuffdiff data for gene lists identified by ChIRP and to generate plots for figures (27,43). The chi-squared test used to identify the enrichment of significantly changed genes overlapped between Zt6 WT/*Snord116del* changed genes and the ChIRP-seq gene set (*P* = 2.2 × 10^−16^), the diurnally regulated gene set (*P* = 2.2 × 10^−16^) and the WT Zt6/Zt16 gene set (*P* = 2.2 × 10^−16^).

### Metabolic chamber analysis

Mice were placed in Oxymax/CLAMS (Columbus Instruments) at the UC Davis Mouse Metabolic Phenotyping Center and monitored for 7 days with six full 24-h light/dark cycles. Only male *Snord116del^+/−^* mice were analyzed by CLAMS. Locomotion, food and water intake, O_2_ consumption and CO_2_ emission were automatically monitored. To allow the acclimation of mice to CLAMS chambers only the final four full light/dark cycles were used in analysis of energy expenditure. Significant changes tested by two-tailed Student's *t*-test.

### Body weight and percent fat

After being removed from CLAMS chambers, mice were weighed, anesthetized with isofluorane and scanned using dual energy X-ray absorptiometry (Piximus, Fitchburg WI, USA).

### qPCR

qPCR analyses were performed using Sybr Green (Bioline) on an ABi ViiiA 7 in 384-well formats with custom primers (Supplementary Material, Table S7). Significant changes tested by the Mann–Whitney *U*-test.

## SUPPLEMENTARY MATERIAL

Supplementary Material is available at *HMG* online.

## AUTHORS' CONTRIBUTIONS

W.T.P. and J.M.L. designed experiments and wrote manuscript. W.T.P. performed FISH, ChIRP, immunofluorescence, microscopy, sequencing analysis, LSC, qPCR, tissue dissection and CLAMS data analysis. R.L.C. performed FISH, microscopy and tissue dissection. F.K.C. performed mouse husbandry and genotyping, qPCR and RT-qPCR. S.S.W. performed qPCR and RT-qPCR. D.H.Y. designed and performed mouse breeding and phenotyping studies and performed tissue dissection and processing. R.A.A., P.T. and N.A.Y. designed and provided custom OLS FISH probes.

## FUNDING

Work funded by National Institutes of Health (F31NS073164 to W.T.P. and 1R01NS076263 to J.M.L.) and the Prader-Willi Foundation. Funding to pay the Open Access publication charges for this article was provided by Agilent, Inc.

## Supplementary Material

Supplementary Data
