# Supplementary material for: A Prader–Willi locus lncRNA cloud modulates diurnal genes and energy expenditure
Source: Hum Mol Genet. 2013 Jun 13;22(21):4318–28. doi: 10.1093/hmg/ddt281 (PMC3792690; doi:10.1093/hmg/ddt281)
Supplement: Supplementary Data [file supp_22_21_4318__index.html]

A Prader–Willi locus lncRNA cloud modulates diurnal genes and energy expenditure — A Prader–Willi locus lncRNA cloud modulates diurnal genes and energy expenditure — Supplementary Data 

# A Prader–Willi locus lncRNA cloud modulates diurnal genes and energy expenditure

## 

Supplementary Data

**Files in this Data Supplement:**

- Supplementary Data - Pdf file
- Supplementary Tables - xlsx file
