# Supplementary material for: A Prader–Willi locus lncRNA cloud modulates diurnal genes and energy expenditure
Source: Hum Mol Genet. 2013 Jun 13;22(21):4318–28. doi: 10.1093/hmg/ddt281 (PMC3792690; doi:10.1093/hmg/ddt281)
Supplement: Supplementary Data [file supp_ddt281_ddt281supp.pdf]

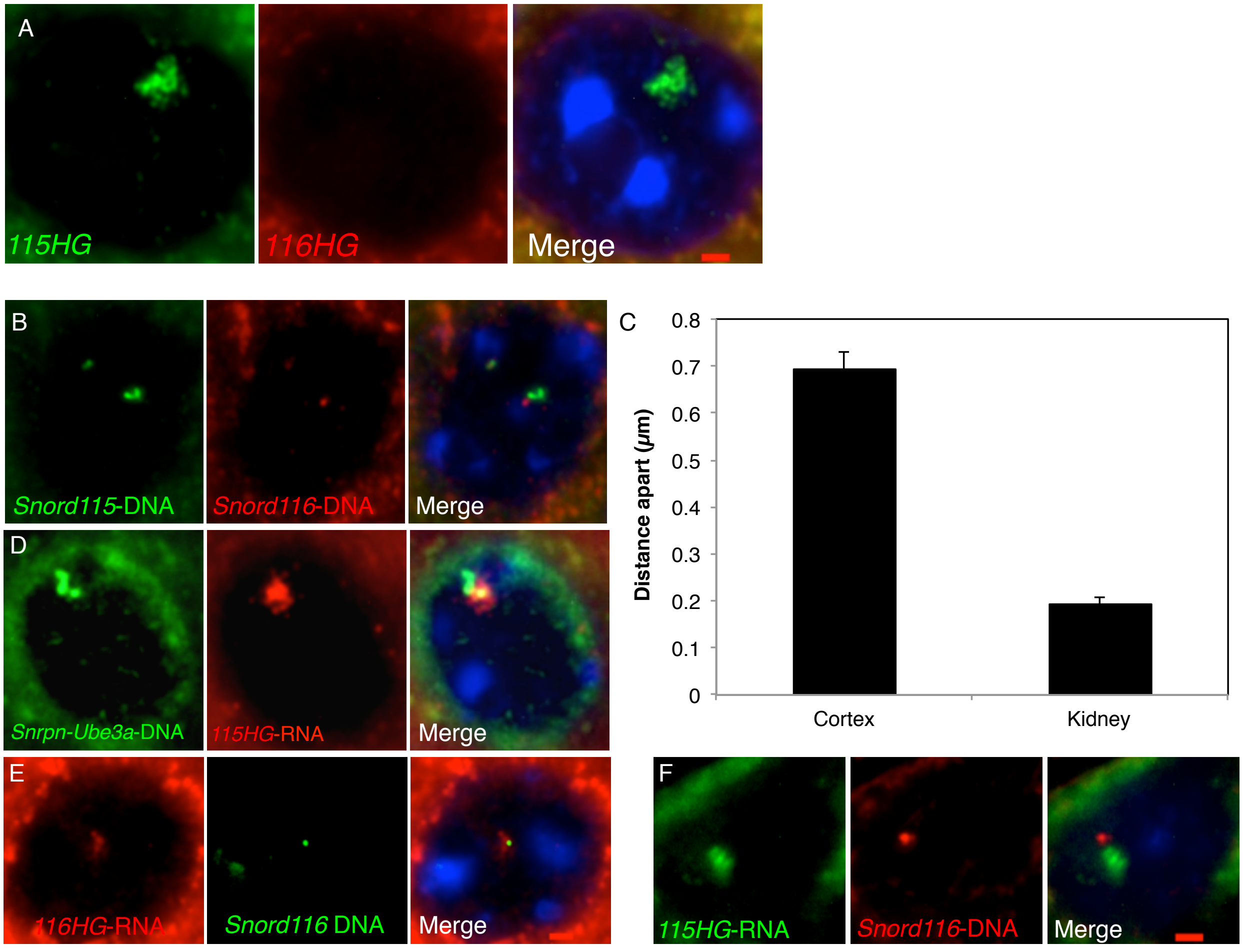

Supplementary Figure S1, related to Figure 2. *116HG* is not present in *Snord116del* (+/-) mice and localizes to the site of its transcription in neuronal nuclei. (**A**) RNA FISH was performed using spliced host gene probes specific for *115HG* (green) and *116HG* (red) on adult wild-type mouse cortex. (**B**) DNA FISH was performed using nick translated BAC probes for *Snord116* (red) and *Snord115* (green) repeat regions on adult wild-type mouse cortex. The decondensed paternal allele in neuronal nuclei shows a separation between the adjacent *116HG* and *115HG* loci. (**C**) Distance between *Snord116* and *Snord115* DNA FISH signals was measured in adult wild-type mouse cortex and kidney nuclei (n=137), showing that chromatin decondensation and separation of the adjacent loci is tissue specific. (**D**) Combined DNA and RNA FISH to adult mouse cortical tissue revealed partial colocalization of the *115HG* lncRNA

(red) with the encoding locus on the paternal decondensed allele of *Snrpn* through *Ube3a* (green). (**E**) The *Snord116* DNA FISH probe colocalized with *116HG*. (**F**) *Snord116* DNA did not colocalize with *115HG*. Nuclei were counterstained with DAPI (blue) and scale bar is 1 μm.

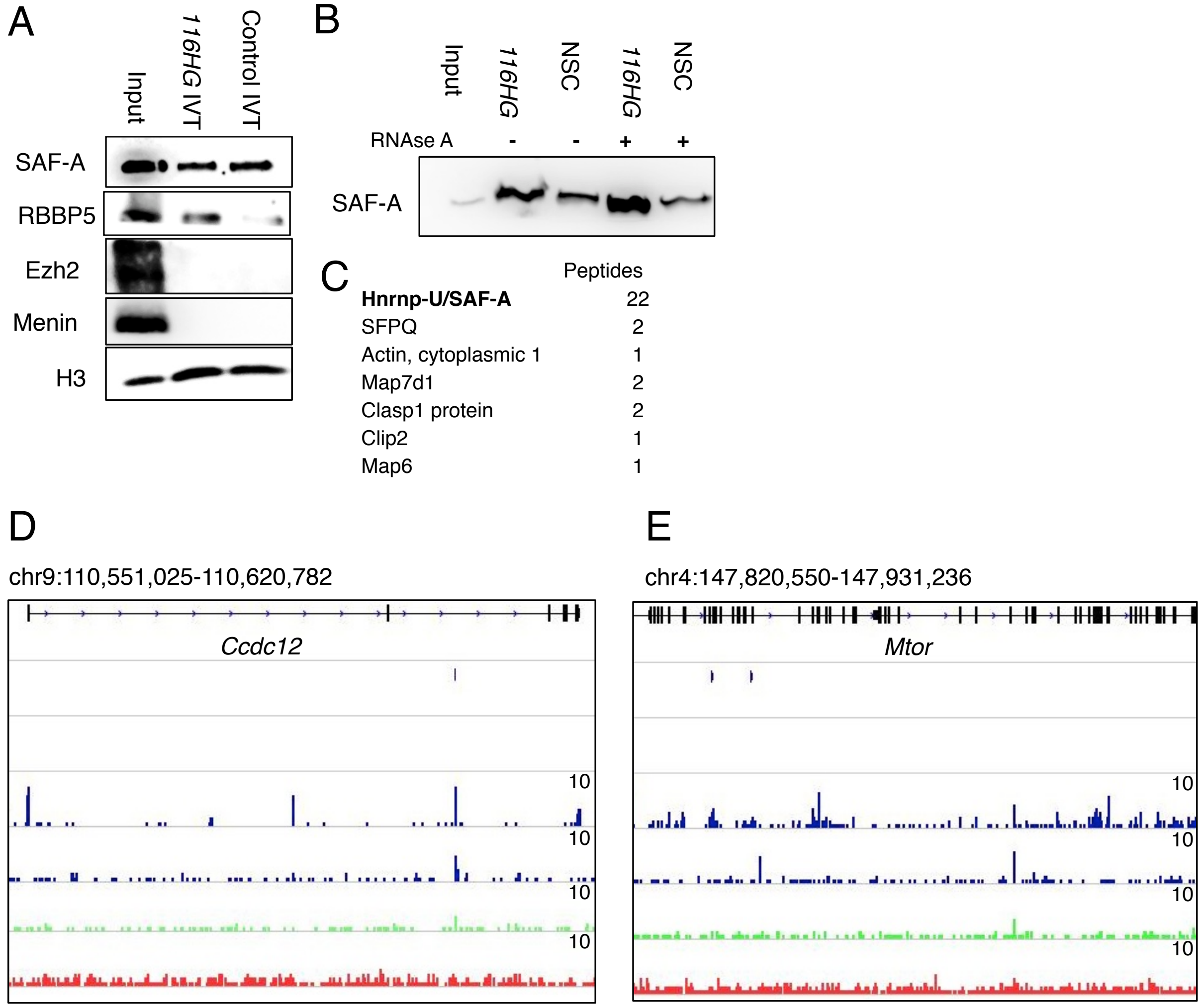

Supplementary Figure S2, related to Figure 2 and Figure 3. SAF-A was identified by mass spectrometry of *116HG* ChIRP, but association was not specific and not RNA dependent, unlike RBBP5. *116HG* is bound to regions genome-wide. (**A**) Pull-down of *in vitro* biotinylated *116HG* retrieved RBBP5, but not Ezh2 or Menin. SAF-A was retrieved by both biotinylated *116HG* and by a biotinylated control IVT RNA. (**B**) ChIRP with *116HG* or non-specific control (NSC) oligos retrieved SAF-A, but the interaction was not reduced with RNAse treatment. (**C**) Mass spectrometry identification of ChIRP-retrieved proteins revealed SAF-A/HNRNP-U as the dominant protein, with 22 peptides. (**D**) and (**E**) Black represents annotated genes. Blue represents peaks of *116HG* ChIRP from WT brain, and read coverage from two independent

ChIRP experiments. Green represents peaks from merge of 3 control ChIRP experiments (*116HG* ChIRP on *Snord116del*$^{+/-}$ brain, NSC ChIRP on WT and *Snord116del*$^{+/-}$ brain) and read coverage from NSC ChIRP on WT brain. Red represents read coverage from WT input. Scale is 0-10 reads.

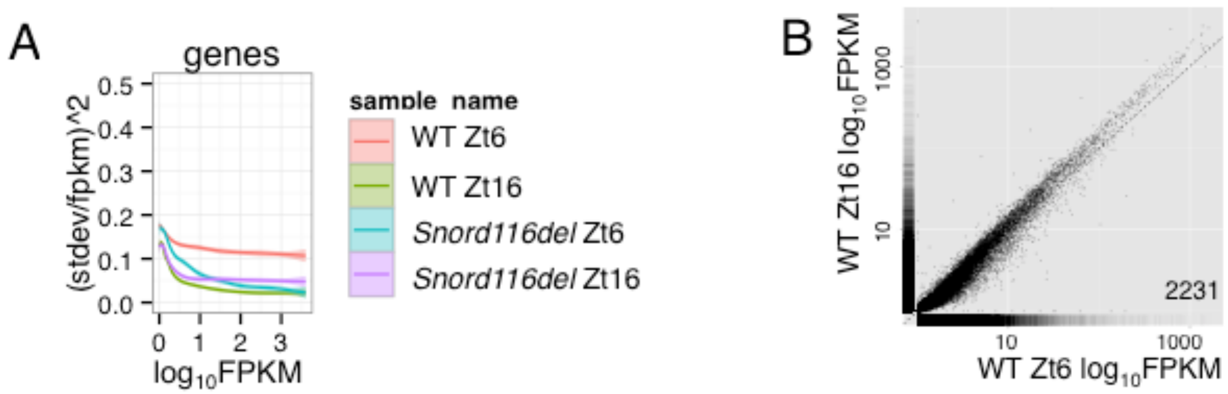

Supplementary Figure S3, related to Figure 5. RNA-seq reveals time-of-day dependent genotype effects on transcript levels (**A**) CummeRbund generated plot of squared coefficient of variation of all samples used in RNA-seq analysis. (**B**) Comparison of expression in WT mice by RNA-seq shows 2,231 genes changed with time of day.

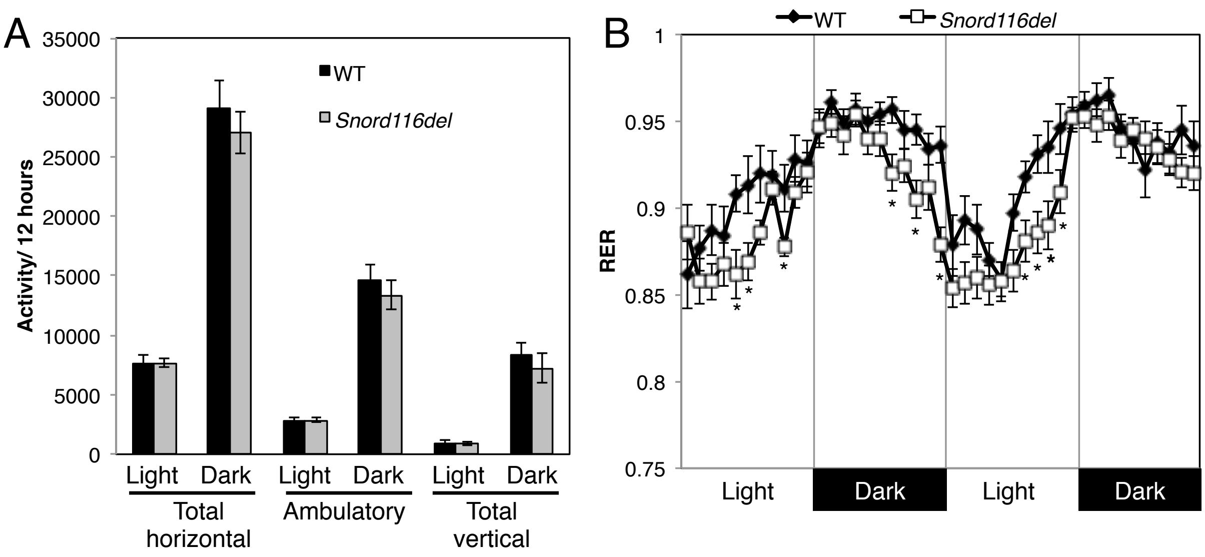

Supplementary Figure S4, related to Figure 6. *Snord116del*$^{+/-}$ are smaller with less fat mass and have normal rhythms of food intake and activity. (**A**) *Snord116del*$^{+/-}$ mice do not differ from WT littermates in total horizontal, ambulatory, or total vertical activity. (**B**) *Snord116del*$^{+/-}$ mice exhibit lower RER during light hours as compared to WT mice, but do have diurnal cycling of RER.

Supplementary Tables 1-7 as separate excel file.
